# Admission of a Terminally Ill Lung Cancer Patient With the Accidental Diagnosis of SARS-CoV-2 to a Palliative Care Unit Resulting in a SARS-CoV-2 Outbreak

**DOI:** 10.1177/21650799211042518

**Published:** 2021-12-13

**Authors:** Carmen Roch, Ulrich Vogel, Katharina Smol, Steffen Pörner, Birgitt van Oorschot

**Affiliations:** 1 University Hospital Würzburg; 2 University of Würzburg

**Keywords:** SARS-CoV-2 outbreak, palliative care, respirators, room ventilation

## Abstract

The COVID-19 pandemic poses challenges for palliative care. Terminal patients cannot wear masks and may demonstrate unspecific symptoms reminiscent of those caused by COVID-19. This report is about a terminally ill patient with lung cancer who displayed fever, cough, and fatigue. During hospital admission screening, the patient tested negative for SARS-CoV-2. When admitting his wife to stay with him, she also had to test for SARS-CoV-2 and displayed a positive test result. Until the positive results were reported, six staff members were infected with SARS-CoV-2, even though they were routinely wearing respirators. This resulted in the palliative care unit having to be closed. Hospitals need strict and adequate testing and re-testing strategies even for intra-hospital transfers. Workers must strictly adhere to recommended respirator practices. Ventilation of patient rooms is essential due to the possible enrichment of particle aerosols containing viruses, as negative pressure rooms are not recommended in all countries.

## Background

The novel coronavirus disease (COVID-19) has spread rapidly around the world, posing major challenges to health systems and all health care providers (Pollard et al., 2020). During the pandemic, patients often died in hospitals without being accompanied by their relatives or palliative care specialists (Ingravallo, 2020; Moore, 2020). Palliative care specialists fought hard to allow patients to be accompanied and tried to relax the often strict visitor bans (“Palliative Care and the COVID-19 Pandemic,” 2020). However, to permit visits, infection control measures required both visitors and patients to wear masks (Leung et al., 2020). It was not feasible to obligate terminally ill or dying patients to wear medical masks to reduce the spread of disease due to several physiological changes that caused them discomfort (Li et al., 2005; Purushothaman et al., 2020). Source control on the side of the patient is hampered by the fact that patients are often bedridden and require assistance from caregivers for many daily activities, including positioning, which involves activities close to the body. For this reason, it was necessary for staff to protect themselves by using respirators that filtered aerosolized particles (Cook, 2020; Fouladi Dehaghi et al., 2020). COVID-19 frequently occurs in larger clusters due to superspreading events (Adam et al., 2020). Despite the characteristic overdispersion of SARS-CoV-2 (Endo et al., 2020), outbreaks in palliative care units have not yet been reported to the best of our knowledge.

## Case Presentation

Our inhouse specialist palliative care service (PCS) treated a 75-year-old man suffering from lung cancer. Originally, the patient was transferred from an orthopedic clinic where a pathological lumbar fracture was diagnosed. The patient was transferred to our internal medicine unit for further tumor examination. At the time of cancer diagnosis in November 2020, adenocarcinoma of the right upper lobe of the lung with osseous and lymphogenic metastases was far advanced, as well as metastases of the adrenal gland. Due to a rapidly worsening state of health and aggravating symptoms, such as dyspnoea and weakness, the planned radiotherapy and commencement of a tumor-specific therapy could not be carried out. Therefore, PCS management was initiated. During the patient’s hospital stay, his wife was his only visitor due to COVID-19 visitation restrictions. Before beginning PCS care, the patient had already been hospitalized for approximately 3 weeks in the internal medicine unit of the hospital, and an oropharyngeal swab for a SARS-CoV-2 PCR (polymerase chain reaction) test had last been performed on the day of admission to the hospital with a negative result.

On the day of transfer to the palliative care unit, the patient’s symptoms became significantly more severe. He suffered increasingly from shortness of breath and a cough, as well as developing a fever. Visitors were required to fill out a questionnaire before every visit asking about any potential contact with COVID-19 infected individuals and symptoms of infection, but a measurement of body temperature was not obtained. On admission to our palliative care unit, the patient’s wife denied that she or her husband had contact with anyone infected with COVID-19, and according to her questionnaire answers, she was free of symptoms. The patient’s symptoms were interpreted as being due to pneumonia from pre-existing lung cancer. Wearing a mask was impossible for the patient. The nurses along with other members of our multiprofessional palliative care team (social worker, psychologist, chaplains, physiotherapist, and doctors) used filtering face pieces class 2 (FFP2) tested according to the norm EN 149. The performance of FFP2 masks is mostly comparable with that of N95 respirators. However, the team members did not wear face shields or protective gowns at the bedside. Two days after transfer, the patient’s condition deteriorated, and his wife moved in to accompany her husband during the terminal phase. According to the hospital’s rules, relatives who are admitted must have an oropharyngeal swab for SARS-CoV-2 PCR. A few hours after the wife’s admission, the laboratory informed us that she was positive for SARS-CoV-2 with a cycle threshold value (ct value) of 15.5. Therefore, an oropharyngeal swab was also performed on the patient, which was positive test (ct value of 24). The patient’s wife was sent home for self-isolation in accordance with public health requirements.

The cancer patient died 4 hours after his wife went home. A report was made to the infection control team in cooperation with the hospital’s occupational health department to inform them of the patient’s and his wife’s infection with SARS-CoV-2. They ordered that all palliative care staff who worked during the couple’s stay on the ward be tested. To our knowledge, no other patients on the ward were positive for COVID-19, nor were other sources of infection identified.

**Figure 1.**
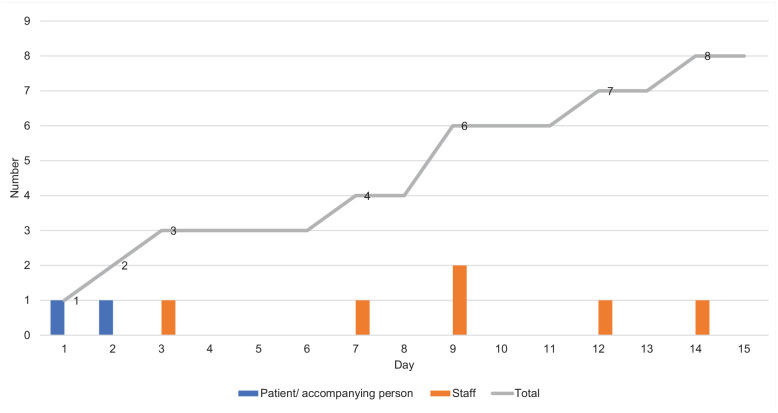
Distribution of a SARS-CoV-2 outbreak in the palliative care unit originating from an infected patient and an accompanying person.

A COVID-19 PCR screening was performed on 18 staff members, irrespective of the type of contact protection measures they had been using. Most (*n* = 14) staff members were identified as having direct contact with the patient and/or wife. Testing was repeated every second day for 14 days so as to identify potentially infected staff during the COVID-19 incubation period. During this 14-day period, six staff members tested positive for SARS-CoV-2 (Figure 1). In collaboration with the infection control team, the department decided to close the unit. This was necessary, first, to terminate the outbreak of SARS-CoV-2 and, second, because there was a shortage of staff.

Respirator use was a key component of the infection prevention protocol. Donning and doffing respirators, as well as conducting appropriate seal checks, had been taught by infection control nurses in collaboration with occupational health in line with internal standards. All infected team members indicated they were confident that they had worn the mask properly. There is, however, no legal obligation in Germany to regularly conduct respirator fit tests. As a result, these tests were not conducted by occupational health prior to the outbreak. Re-using or re-processing respirators was not encouraged at the hospital.

## Discussion

This case report demonstrates that re-testing patients and accompanying persons regularly after admission and before intra-hospital transfer would have prevented or mitigated the development of the outbreak among our staff. The patient had tested negative on admission to the internal medicine unit, but no re-testing was done on transfer to the palliative care unit. Clinical signs of COVID-19 such as cough, fatigue, and fever, especially in palliative care patients, might be misinterpreted and diagnosis may therefore be delayed, which again argues for repeated testing (Harada et al., 2020; Huang et al., 2020; Pulia et al., 2020). Following the outbreak, re-testing of patients was initiated on Day 4 of the hospital stay.

The palliative care unit is located in a building constructed in the 1960s. There is a ventilation system; however, air exchange rates are very low, and there are no positive pressure ventilation rooms. Very early on, the hospital’s incident command group decided, according to federal recommendations, that COVID-19 patients may only be cared for outside of wards designated for COVID-19 patients in well-defined exceptions and based on risk assessment. In this particular case, the reason for not transferring the patient to the ward for infectious patients was the fact that he was already dying.

The large number of workers who were exposed suggests that the poor ventilation situation in the patient’s room may have fostered the outbreak due to a high viral load in the air. The patient’s room was a normal Class II ward, for which there are no special air hygiene requirements for non-infectious patients according to German recommendations (Christiansen et al., 2015). Opening the windows on a regular basis and preferably always before staff contact with patients requires high discipline and is frequently viewed as uncomfortable by patients, especially during winter. This particular room, with an air exchange rate of 1 room volume per hour, was most likely not providing sufficient air volume exchanges. Ventilation by means of regularly opening windows has now been introduced in accordance with the strict ventilation concepts required for SARS-CoV-2 prevention (Tang et al., 2021). A recent study underlined the importance of ventilation by demonstrating how there was a significant difference in air contamination with SARS-CoV-2 in household rooms compared with patient rooms, which could be explained by the fact that only the latter were actively ventilated (Munoz-Price et al., 2021).

Staff informed the infection control team that they wore respirators during the entire work shift; however, we did not conduct surveillance to measure adherence. Wearing respirators without practicing correct donning and doffing or performing proper seal checks may provide a false sense of security as they are supposed to reduce the risk of infection (Iannone et al., 2020); however, the effectiveness of a respirator strongly depends on how closely the mask fits the contour of the face (Regli et al., 2021). Although infections by both airborne and droplet transmission are significantly reduced by the continuous wearing of respirators, infections may still be detected, at least for influenza (MacIntyre et al., 2017). As the correct fit and wearing of the respirator were checked in the studies and the staff were instructed and trained in this regard, the results can only be transferred to everyday clinical practice to a limited extent (MacIntyre et al., 2017). It may, therefore, not be surprising to see outbreaks in health care facilities despite continuous respirator use (MacIntyre & Chughtai, 2020; MacIntyre et al., 2017). Consequently, respirators should only be used after successful fit testing and training on proper donning and doffing according to Occupational Safety and Health Administration (OSHA) Standard 1910.134 App B-1 (User Seal Check Procedures; U.S. Department of Labor, 1998), which currently is not the standard at many institutions including our own. Furthermore, according to international recommendations, they should be used in defined situations, such as when taking care of COVID-19 patients, during exposure to aerosol transmissible diseases, or while in close contact with patients who are not capable of wearing a mask, which, in turn, hampers source control (Centers for Disease Control and Prevention, 2020).

## Conclusion

In summary, the risk of COVID-19 infection will only be reduced if protective measures and tests are consequently performed, even with regard to palliative care patients who need to have contact with their relatives. Furthermore, it is necessary to both teach and practice how personal protective equipment should be correctly fitted and used. Only by being aware of the correct fit; using a respirator, gown, and face shield; and employing appropriate ventilation concepts can we mitigate the risk of transmission. Regular training to demonstrate donning and doffing the protective equipment should be provided. Wherever possible, COVID-19-patients should be placed in negative pressure rooms with high air exchange rates. Furthermore, during high incidence periods, regular PCR testing of patients even after the initial admission screening should be conducted.
